# Comparison between the Impacts of Memory and Arithmetic-Based Dual Tasks on Physical Performance in Women with Fibromyalgia

**DOI:** 10.3390/biology11070947

**Published:** 2022-06-21

**Authors:** Jesús Sánchez-Gómez, Santos Villafaina, Francisco Javier Domínguez-Muñoz, Juan Luis Leon-Llamas, Alvaro Murillo-Garcia, Narcis Gusi

**Affiliations:** 1 Universidad de Extremadura, Facultad de Ciencias del Deporte, Grupo de Investigación Actividad Física y Calidad de Vida (AFYCAV), 10003 Caceres, Spain; jesusssg6@gmail.com (J.S.-G.); fjdominguez@unex.es (F.J.D.-M.); alvaromurillo@unex.es (A.M.-G.); ngusi@unex.es (N.G.); 2 Departamento de Desporto e Saúde, Escola de Saúde e Desenvolvimento Humano, Universidade de Évora, 7004-516 Évora, Portugal; 3 International Institute for Innovation in Aging, University of Extremadura, 10003 Caceres, Spain

**Keywords:** dual task, pain, physical fitness tests, flexibility, strength, activities of daily living

## Abstract

**Simple Summary:**

This article focuses on comparing two types of cognitive tasks (memory or arithmetic) while performing a physical task. The application of two simultaneous tasks is known as the dual-task paradigm. In fibromyalgia, its analysis has become of great interest since most of the activities of daily living involve physical and cognitive tasks that are impaired in this disease. The physical fitness tests chosen for this study took into account the flexibility, strength, and movement patterns involved in activities of daily living. The simultaneous cognitive tests were: (1) Immediately before the test, three random words had to be memorized by participants, and immediately after the test these words had to be recalled aloud (memory task); and (2) counting aloud backward in rows of two, starting from a random number between 50 and 100 (arithmetic task). The results showed that the arithmetic task had a more significant impact on physical performance than the memory task on the upper-extremity flexibility and lower-extremity strength of women with fibromyalgia. In addition, cognitive performance was lower in the arithmetic- than in the memory-based task. The characteristics of the sample, environment, complexity of the motor task, and difficulty of the simultaneous cognitive task may also be relevant for understanding the differences in interference caused by the two types of cognitive task (arithmetic or memory).

**Abstract:**

Background: Fibromyalgia symptoms have a significant impact on the ability to perform activities of daily living. These activities require the ability to perform two or more tasks at the same time, which is known as a dual task. Purpose: To analyze physical and cognitive performance differences between memory and arithmetic dual tasks. Methods: Twenty-five women with fibromyalgia participated in this study. Participants performed three physical tests (back scratch, arm curl, and 10-step stair) as a single task and under two types of dual task (memory and arithmetic). Results: Differences between the single and dual tasks were observed in the back scratch and the 10-step stair tests using the arithmetic dual-task. Significant differences were only observed for the memory dual-task in the 10-step stair test. In addition, the performance in the back scratch and in the 10-step stair was significantly lower under the arithmetic compared to the memory-based dual task. Furthermore, a significant difference between these two types of dual task was obtained in the dual-task cost of 10-step stair. Regarding cognitive performance, a significantly lower percentage of correct responses was found in the AbDT compared to the MbDT in the 10-step stair test. Conclusions: the AbDT could have a higher impact on physical performance than the MbDT during the back scratch and the 10-step stair tests. The characteristics of the sample, environment, complexity of the motor task, and difficulty of the simultaneous cognitive task may also be relevant for understanding the differences in dual-task interference.

## 1. Introduction

Fibromyalgia (FM) is a chronic disease characterized by widespread, persistent pain and other symptoms, such as sleep disorders, anxiety, depression, stiffness, fatigue, balance, or mobility problems [1,2]. The estimated prevalence is around 2% to 3% worldwide [3], with women more affected (>90%) than men [4,5]. In this regard, previous investigations reported cognitive impairments in several domains, including processing speed [6], short-term memory [7], long term memory [8], inhibitory control [9], and working memory [10].

In daily life, activities are usually presented as two or more tasks simultaneously [11]. This is known as the dual-task paradigm (DT). In this regard, Lacour, et al. [12] explored the different DT models according to how one task could affect the performance of the other task. Therefore, three models were observed: (1) the cross-domain competition model (where tasks compete for attentional resources); (2) the U-shaped interaction model (performance can be improved or diminished depending on the difficulty of the secondary task); and (3) the task prioritization model (postural control would be prioritized over the cognitive task in older adults). Previous studies in FM have studied the impact of adding a cognitive task on physical performance [13,14,15,16,17]. In this regard, the physical performance is generally reduced when a cognitive task is added in women with FM [13,15,17]. Moreover, de Gier, Peters, and Vlaeyen [13] observed that the pain at baseline would determine physical performance. Moreover, during DT conditions, pain perception seemed to be attenuated; this occurred also when the difficulty of the task increased [18,19,20,21]. This could be due to the bidirectional interaction between attention and pain processing that depends on the relative ability to capture limited attentional capacity resources [22]. This suggests that performing a more demanding task distracts attention from pain perception [21]. This is relevant since kinesiophobia (fear of movement due to painful feelings) is prevalent among people with FM [23].

Different types of DT conditions have been proposed in women with FM, for instance, counting backwards [24] or remembering three unrelated words [14] while performing a motor task. Nevertheless, not all tasks seem to have the same impact on physical performance. For example, Villafaina et al. [14] reported that a memory-based DT seemed to be too easy to cause interference. Therefore, the investigation of other types of DT condition is needed to find out which type causes more interference. Thus, this study aimed to: (1) compare the physical and cognitive performances of two types of DT (a memory-based DT and an arithmetic-based DT), and (2) compare the physical performance between single and DT conditions. Based on the results of previous studies [14,24], we hypothesized that the memory-based DT would less significantly impact physical and cognitive performances than the arithmetic-based DT due to the lower complexity of the memory based-DT. Furthermore, the physical performance during DT would be reduced when compared to the single task condition, as previous studies have reported [13,14,15].

## 2. Materials and Methods

### 2.1. Participants

The G*Power software 3.1.9.4 (Kiel University, Kiel, Germany) estimated that a sample size of 19 achieves a 95% power to detect significant differences with an alpha of 0.005, using the Wilcoxon signed-rank test. The back scratch data provided by a previous study [24] (−7.45 (9.88) for ST and −12.22 (11.82) for DT with a correlation index of 0.9), was used to make this calculation. Since two measurements were needed, we recruited 30% more participants in case someone decided not to attend the second evaluation. In this case, if someone does not attend the second evaluation, our number of participants would exceed the required sample size. Finally, a total of 25 women with FM (age = 55.28 [10.67] years old and years since FM diagnosis = 10.48 [13.28]) participated in this cross-sectional study (see participants’ characteristics in Table 1). All of them attended the first and second evaluations. The Association of Fibromyalgia (AFIBROEX) recruited the women with FM by telephone call, in April 2018. Inclusion criteria were: (1) diagnosis of FM by a rheumatologist following the criteria of the American College of Rheumatology [1]; (2) be women; (3) not have cognitive impairment according to the Mini-Mental State Examination (MMSE); and (4) be over 18 years old. Participants were excluded if they: (1) were pregnant; (2) had neurological diseases or psychiatric diagnoses or (3) had contraindications for physical activity.

All the participants read and signed the informed consent prior to the first assessment. Procedures were approved by the University bioethical committee (approval number: 62/2017), in accordance with the updated Declaration of Helsinki.

### 2.2. Procedure

Body composition measurements, as well as the impact of the disease, were evaluated before the physical fitness assessment.

Then, participants were informed about the correct development of the physical fitness tests (back scratch test, arm curl test and 10-step stair test). In each session, all the participants performed the physical fitness test in both single task (ST) and one of the following DT conditions:

(1) Memory-based DT (MbDT). Consisted of remembering three random, unrelated words while the women were performing the tests. A list of nine Spanish words was created (corresponding to children, napkin, blanket, euro, doctor, television, arm, boat, and pine tree) and three words were randomly selected for each test. Participants were encouraged to think in these words at the same time the physical test was being completed. Immediately after the tests, participants had to say out the three words and the accuracy was registered. Participants were not required to repeat the DT if they made mistakes in recall.
(2) Arithmetic-based DT (AbDT). Consisted of counting aloud, backwards in rows of two, while performing the tests, starting from a random number between 50 and 100. The number of correct subtractions during the test were registered. Participants were not required to repeat the DT if they made mistakes in subtractions.

The cognitive performance was registered during both MbDT and AbDT. The participants knew that the research team was scoring cognitive performance. However, feedback on performance (both physical and cognitive) was not provided so as not to influence either positively or negatively the development of the following tests.

Conditions (ST and DT), physical fitness tests and types of DT (MbDT and AbDT) were randomized. Furthermore, to avoid the potential learning effect, participants were evaluated twice, two months apart. Therefore, in the first and the second sessions, they performed the MbDT or the AbDT as the randomization dictated. In order to control so that the progression of the disease would not alter the results, the revised version of the Fibromyalgia Impact Questionnaire (FIQr) was administrated at the first and the second evaluations. At the first evaluation, the FIQ mean was 52.51 (17.67), and at the second evaluation, the FIQ mean was 49.39 (18.53). Comparison between first and second evaluations did not show significant differences (*p*-value = 0.235).

### 2.3. Outcomes

#### 2.3.1. Sociodemographic, Body Composition, Cognitive Impairment and Fibromyalgia Impact

Participants were asked about their age and the number of years since FM was diagnosed. A Tanita Body Composition Analyzer BC-418 MA was used to extract the percentage of fat mass and weight. A stadiometer (SECA 225, SECA, Hamburg, Germany) was used to measure participants’ heights, in order to calculate the body mass index (BMI).

In addition, the Spanish version of the MMSE [25] was used to explore if participants had cognitive impairment. This questionnaire includes tests of orientation, attention, memory, language, and visual-spatial skills. The maximum score is 35, and an individual is considered to have a cognitive impartment when the score is <24 for geriatric populations and <28 for a non-geriatric population. Furthermore, the Spanish version of the FIQr was administered to evaluate the impact of the disease [26]. This questionnaire has 21 items scored from 0 to 10 (with 10 representing the worst condition). The FIQr is divided into three domains (function, overall impact, and symptoms). The maximum score is 100, which corresponds to the worst overall impact of symptoms.

#### 2.3.2. Back Scratch Test

The back scratch test has been previously used to assess the flexibility of the upper limbs in people with FM [27]. Participants had to try to touch with the middle finger of both hands behind the back. Thus, they passed one hand behind the head, flexing the elbow and directing the middle finger down while they made the opposite procedure with the other hand. For the analysis, the mean between the two was chosen.

#### 2.3.3. Arm Curl Test

The arm curl test has been previously used to assess the strength of upper limbs in people with FM [27]. The participants were seated in a chair, holding a 2.5 kg weight. They had to start with the elbow fully extended. Then, participants had to bend their elbows to lift the weight and return to the starting position as many times as possible for 30 s. The test was performed with both dominant and non-dominant arms. For the analysis, the best of the two trials for each arm was chosen.

#### 2.3.4. 10-Step Stair Test

The 10-step stair test has been previously used in women with FM, and it is related to lower limb strength [28]. Participants had to climb ten stair steps without hand-rails and at a self-selected safe pace. The start was static, and the test ended when participants arrived at the last stair-step. The time participants expended climbing the 10-step was recorded.

#### 2.3.5. Cognitive Performance

Cognitive performance in the MbDT was assessed counting the number of errors and correct words recalled by the participants. In the AbDT, the total number of operation, errors, and correct responses were recorded.

### 2.4. Statistical Analysis

The SPSS statistical package (version 20.0; SPSS, Inc., Chicago, IL, USA) was used to analyze the data. Based on the results of Shapiro-Wilk and Kolmogorov-Smirnov tests, non-parametric tests were performed.

Wilcoxon signed-rank tests were conducted to examine differences between the two types of DT (MbDT and AbDT) as well as to analyze differences between ST and DT in the MbDT and AbDT. Moreover, the dual-task cost (DTC) was calculated for each type of DT. This is calculated as follows:

DTC = (result of DT condition–result of ST condition)/result of ST condition.

Therefore, the Wilcoxon signed-rank test was also used to examine the differences between the DTC of the two types of DT.

Effect size, r, was calculated for each of the comparisons [29]. Values of 0.37, 0.24, and 0.10 represent large, medium, and small effect sizes, respectively [30]. The alpha level of significance (0.05) was adjusted according to the Benjamini–Hochberg procedure to avoid type I error derived from multiple comparisons [31].

## 3. Results

Table 2 shows the differences between ST and DT conditions. Using the MbDT, a significant difference was only observed in the 10-step stair test (*p*-value = 0.009). Moreover, differences between ST and DT using the AbDT were obtained in the back scratch (*p*-value < 0.001) and 10-step stair (*p*-value < 0.001) tests.

Table 3 shows the differences between the MbDT and AbDT, as well as the performance in ST condition, for each physical fitness tests in the two evaluation sessions. Significant differences were not observed between ST in the two evaluations for any of the physical fitness tests (*p*-value > 0.05).

Regarding the DT condition, significant differences were observed between the MbDT and AbDT performances in the DT condition (*p*-value = 0.003) in the back scratch. In this regard, a worse performance was observed in the AbDT than in the MbDT condition. Nevertheless, the DTC in the back scratch did not statistically differ between these two conditions (*p*-value = 0.987). In the arm curl test, significant differences were not obtained in the DT nor the DTC (*p*-value > 0.05). Lastly, in the 10-step stair test, significant differences were observed between DT conditions (*p*-value = 0.018) and DTC (*p*-value < 0.001). In this regard, as observed in the back scratch test, a worse performance was obtained in the AbDT than in the MbDT condition.

Table 4 shows the cognitive performances in the two types of DT (MbDT and AbDT). Although descriptive data were reported for errors and correct responses, statistical analyses were only conducted to take into account the percentage of correct responses, since different scale ranges were used in the MbDT and AbDT. A significantly lower percentage of correct responses was found in the AbDT compared to MbDT in the 10-step stair test (*p*-value = 0.005). Significant differences in cognitive performance were not found between MbDT and AbDT involving the back scratch test and the arm curl test.

## 4. Discussion

The main purpose of this study was to compare two types of DT: one focused on a secondary task based on memory and another focused on arithmetic, while women with FM performed three physical fitness tests. Results showed that the AbDT had a higher impact on the physical performance than the MbDT in the back scratch and 10-step stair test of women with FM. Furthermore, this study also aimed to compare the physical performances between ST and DT conditions. Results showed significant differences between ST and DT in the back scratch and 10-step stair alongside the AbDT. Moreover, using the MbDT, a significant difference between ST and DT was observed only in the 10-step stair test. In addition, a significantly lower percentage of correct responses was found in the AbDT compared to MbDT in the 10-step stair test (*p*-value = 0.005).

DT performance in women with FM is relevant since this population has reported a reduced ability to perform the activities of daily living (which are usually presented as DT conditions, such as walking while having a phone conversation, or climbing stairs while thinking about personal problems, etc.). Unfortunately, women with FM have shown reduced DT performances when compared with healthy controls in physical fitness tests [14,15], postural control [13], or balance [32]. Villafaina, Collado-Mateo, Domínguez-Muñoz, Fuentes-García, and Gusi [14] showed that differences between ST and DT were higher in the 10-step stair test than in the arm curl test or the handgrip strength test. This is in line with our results, where we observed that the AbDT condition produced a significant decrement in performance in the 10-step stair test and the back scratch, which was not observed in the arm curl test. Moreover, a significant difference between MbDT and AbDT was also observed in the 10-step stair test DTC.

Regarding task complexity, McIsaac, et al. [33] proposed a taxonomy to help classify each task along broadly identified task characteristics. This categorization can classify tasks as low and high complexity and as low and high novelty. This creates a framework to categorize overall activity as “easier”, “moderate”, or “harder”. In addition, task constraints and the environmental context must be considered [33,34]. In this regard, people with FM showed physical impairments, with lower strength or higher risk of falls, among others deficits [35]. Therefore, taking into account the previously cited framework and the characteristics of people with FM, the 10-step stair test might be considered of high difficulty for this population, whereas the back scratch and the arm curl might be considered of low difficulty in the ST condition. Regarding the simultaneous cognitive task, the MbDT required short-term memory, memory encoding, and retrieval processes [36,37] and AbDT required working memory and performing mental manipulation [38,39]. Some of these cognitive skills have been found to be altered in people with FM [40,41]. These cognitive characteristics could render MbDT and AbDT to be considered highly difficult. However, considering a previous study focused on women with FM [14], in the MbDT, three unrelated words are probably not enough to be considered highly complex. In this regard, more words (five to seven, as used in a previous study [42] would be needed to consider this task as highly complex. Therefore, MbDT in the back scratch and arm curl can be classified as easier tasks, and the 10-step stair test as a moderately difficult task. In contrast, the back scratch test and arm curl test using the AbDT can be classified as a moderate difficulty, whereas the 10-step stair tests are classified as harder difficulty. This framework could explain why greater interferences were found in the 10-step stair test.

Taking into account our results, AbDT seemed to have a greater impact on physical performance than MbDT. Previous studies have observed that women with FM often have attention deficit disorder [43,44] and working memory impairment [40,45], which might lead to difficulties in focusing their attention on the three unrelated words provided before the test started. However, the percentage of correct responses during AbDT conditions ranged from 95.83 (14.95) to 97.33 (9.23), which can reveal a ceiling effect of this cognitive task. In contrast, the MbDT exhibited a broader pattern, with a percentage of correct responses that ranged from 79.23 (30.71) to 93.15 (10.45). In this sense, significant differences in correct response percentages were found in the 10-step stair test between MbDT and AbDT, with a significantly lower percentage in the AbDT. These findings are in line with those obtained for the physical performance, where differences in the DTC and DT (in the back scratch test and 10-step stair test) between MbDT and AbDT were found. According to the conceptual model for characterizing patterns of cognitive-motor, dual-task interference proposed by Plummer and Eskes [45], these results could mean that performing the AbDT with the 10-step stair test induced mutual interference. According to Plummer and Eskes [45], mutual interference suggests the presence of inadequate attentional resources to maintain single-task-level performance in each task when conducted at the same time. Unfortunately, ST performance was not registered for the cognitive task; thus, this hypothesis cannot be confirmed. Nevertheless, this information could be relevant for future studies or physical exercise interventions that include DT exercises in their programs.

In the MbDT, participants were asked to recall three unrelated words immediately before the test. Participants were encouraged to continue thinking of these words at the same time the physical test was being completed. However, taking into account the above-mentioned characteristics of women with FM (attention deficit disorder or working memory impairment), this could mean that women with FM “forgot” to continue thinking of the three unrelated words during the DT condition assessment. Nevertheless, results from the 10-step stair test under the MbDT condition showed a significantly lower physical performance in the DT condition than in the ST condition. This could indicate that participants were processing during the physical fitness test. In order to avoid this issue, previous studies have used a different approach, asking people to recall words during the physical test instead of immediately after [42,46]. In this regard, Goh, Pearce, and Vas [42] compared four types of different DT, including counting backwards and recalling a list of words. Results showed that the AbDT was more demanding (with higher DTC) than the MbDT. Taking into account the methodology employed by Goh, Pearce, and Vas [42], our results could have confounded the those of our intervention.

A previous study showed that when participants were distracted during painful stimulation, the brain pain matrix (thalamus, insula, or anterior cingulate cortex) reduced its activation [47]. Thus, DTs have been used as distractions from pain in chronic pain populations [48]. In this regard, previous studies have shown that the greater the difficulty of the task, the lower the pain perception during the task [18,19,20,21]. This is especially interesting in people with FM since kinesiophobia (fear of movement due to painful feelings) is prevalent [23]. Thus, AbDT could be used during exercise intervention to distract from pain, as well as to achieve higher volumes of physical activity than traditional exercise interventions.

This study has some limitations that should be mentioned. The relatively small sample size (N = 25) could mean that only greater differences would reach the significance level. Moreover, all the participants were women, so results cannot be extrapolated to men with FM. Furthermore, evaluations were made two months apart. This could mean that progression or spontaneous recovery of FM symptoms might have influenced the results. However, differences in the impact of disease (FIQr questionnaire) between the first and the second evaluation were not found. In addition, the ST condition for the cognitive task was not registered, so the tradeoffs and the prioritization frameworks proposed by Plummer and Eskes [45] cannot be confirmed.

## 5. Conclusions

The AbDT could have a higher impact on physical performance than the MbDT in the back scratch test and the 10-step stair test in women with FM. Furthermore, in the 10-step stair test, significantly lower cognitive performance during MbDT was observed than in the AbDT. The characteristics of the sample, environment, complexity of the motor task, and difficulty of the simultaneous cognitive task may also be relevant for understanding the differences in the interference caused by the two types of secondary cognitive tasks.

## Figures and Tables

**Table 1:** Participants’ characteristics.

Measurements	Mean (SD)
Sample size (N)	25
Age (years)	55.28 (10.67)
Weight (kg)	70.08 (10.73)
Fat mass (%)	24.06 (9.84)
BMI (kg/m^2^)	27.37 (3.87)
FIQr total score	52.51 (17.67)
MMSE	32.76 (2.20)
Years since diagnosis	10.48 (13.28)
Years with FM symptoms	18.48 (7.08)

BMI: Body mass index; SD: Standard deviation; FIQr: Fibromyalgia impact questionnaire; FM: Fibromyalgia; MMSE: Mini Mental State Examination.

**Table 2:** Differences between single and dual-task conditions using memory-based and arithmetic dual tasks.

Outcomes	ST Median (IQR)	DT Median (IQR)	Z	*p*-Value	Effect Size
Back scratch test					
MbDT back scratch (cm)	−6.00 (15.88)	−7.75 (14.63)	−0.885	0.376	0.171
AbDT back scratch (cm)	−5.50 (15.00)	−11.00 (18.00)	−4.019	<0.001 *	0.804
Arm curl test					
MbDT arm curl (rep)	17.50 (5.50)	16.75 (4.80)	−1.029	0.303	0.206
AbDT arm curl (rep)	16.00 (3.80)	15.00 (4.00)	−1.571	0.116	0.314
10-step stair test					
MbDT 10-step stair test (s)	4.65 (1.57)	5.05 (1.80)	−2.631	0.009 *	0.526
AbDT 10-step stair test (s)	4.34 (1.34)	5.27 (2.06)	−4.167	<0.001 *	0.833

* p-value < 0.05. DT: Dual task; DTC: Dual-task cost; IQR: Interquartile range; ST: Single Task.

**Table 3:** Performance of women with fibromyalgia in a single task and during two types of dual-task conditions.

Outcomes	Memory-Based DT Median (IQR)	Arithmetic-Based DT Median (IQR)	Z	*p*-Value	Effect Size
Back scratch test					
ST back scratch (cm)	−6.00 (15.88)	−5.50 (15.00)	−1.009	0.668	0.202
DT back scratch (cm)	−7.75 (14.63)	−11.00 (18.00)	−3.281	0.003 *	0.656
DTC	0.05 (0.50)	0.16 (0.60)	−0.016	0.987	0.003
Arm curl test					
ST arm Curl (rep)	17.50 (5.50)	16.00 (3.80)	−0.539	0.668	0.108
DT arm Curl (rep)	16.75 (4.80)	15.00 (4.00)	−1.330	0.184	0.266
DTC	−0.04 (0.16)	−0.06 (0.36)	−1.257	0.313	0.251
10-step stair test					
ST 10-step (s)	4.65 (1.57)	4.34 (1.34)	−0.429	0.668	0.086
DT 10-step (s)	5.05 (1.80)	5.27 (2.06)	−2.500	0.018 *	0.500
DTC	0.04 (0.06)	0.16 (0.18)	−3.600	<0.001 *	0.720

* p-value < 0.05. DT: Dual task; DTC: Dual-task cost; IQR: Interquartile range; ST: Single Task.

**Table 4:** Cognitive performance of women with fibromyalgia in the two types of dual-task conditions.

Outcomes	Memory-Based DT Mean (SD)	Arithmetic-Based DT Mean (SD)	Z	*p*-Value	Effect Size
Back scratch test					
Error (n)	0.08 (0.28)	0.72 (1.14)	-	-	-
Corrects response (n)	2.92 (0.28)	6 (3.54)	-	-	-
Percentage of correct response (%)	97.33 (9.23)	87.17 (20.22)	−1.808	0.071	0.362
Arm curl test					
Error (n)	0.12 (0.45)	0.80 (1.26)	-	-	-
Corrects response (n)	2.88 (0.45)	13.40 (5.11)	-	-	-
Percentage of correct response (%)	95.83 (14.95)	93.15 (10.45)	−1.007	0.314	0.201
10-step stair test					
Error (n)	0.12 (0.44)	0.54 (0.72)	-	-	-
Corrects response (n)	2.88 (0.44)	3.42 (1.77)	-	-	-
Percentage of correct response (%)	96 (14.66)	79.23 (30.71)	−2.818	0.005 *	0.564

* p-value < 0.05. DT: Dual task; SD: Standard deviation; ST: Single task.

## Data Availability

The datasets analyzed during the current study are available from the corresponding author on reasonable request.
